# Retraction Note: Uncertainty in estimating the number of contributors from simulated DNA mixture profiles, with and without allele dropout, from Chinese, Malay, Indian, and Caucasian ethnic populations

**DOI:** 10.1038/s41598-022-11821-5

**Published:** 2022-05-04

**Authors:** Kevin Wai Yin Chong, Christopher Kiu‑Choong Syn

**Affiliations:** DNA Profling Laboratory, Biology Division, Applied Sciences Group, Health Sciences Authority, 11 Outram Road, Singapore, 169078 Singapore

Retraction of: *Scientific Reports* 10.1038/s41598-021-84580-4, published online 04 March 2021

The Authors have retracted this Article. After publication of this Article, the Authors were unable to locate consent forms for some of the old pre-2000 samples used for deriving the allele frequencies in this study. If these samples are excluded, the derived STR allele frequencies would be altered which would impact the modelling and affect the conclusions drawn. In view of this, the Authors have retracted this Article to review and revise the data.

Both Authors agree with the retraction and its wording.

